# Acute Effects of Nutritional and Physical Recovery Strategies on Exercise Performance, Muscle Damage, and Fatigue in Elite Basketball Players: A Pilot Randomized Crossover Trial

**DOI:** 10.3390/life16020275

**Published:** 2026-02-05

**Authors:** Alberto Marín-Galindo, Alejandro Perez-Bey, Juan M. Escudier-Vázquez, Daniel Velázquez-Díaz, Julio Calleja-González, Carmen Vaz-Pardal, Juan Corral-Pérez, Jesus G. Ponce-Gonzalez

**Affiliations:** 1 ExPhy Research Group, Department of Physical Education, Instituto de Investigación e Innovación Biomédica de Cádiz (INiBICA), Universidad de Cádiz, 11009 Cádiz, Spain; alberto.marin@uca.es (A.M.-G.); juan.corral@uca.es (J.C.-P.); jesusgustavo.ponce@uca.es (J.G.P.-G.); 2 GALENO Research Group, Department of Physical Education, Instituto de Investigación e Innovación Biomédica de Cádiz (INiBICA), Universidad de Cádiz, 11009 Cádiz, Spain; alejandro.perezperez@uca.es; 3 Fundación Para la Gestión de la Investigación Biomédica de Cádiz, Hospital Puerta del Mar, 9ª Planta, Unidad de Investigación, 11009 Cádiz, Spain; juanmanuel.escudier@uca.es; 4 Physical Education and Sport Department, Faculty of Education and Sport, University of the Basque Country (UPV/EHU), 01006 Vitoria-Gasteiz, Spain; julio.calleja.gonzalez@gmail.com; 5 Andalusian Center of Sport Medicine, Junta de Andalucía, 41092 Cádiz, Spain; carmenvaz@hotmail.com; 6 School of Health Sciences, International University of La Rioja, 26004 Logroño, Spain

**Keywords:** cold-water immersion, recovery strategies, nutrition, team sports, exercise-induced muscle damage

## Abstract

Background: Due to the congested competition calendar and the high physical demands of elite basketball, the selection of effective recovery strategies is essential to optimize performance and reduce exercise-induced fatigue and muscle damage. This pilot study aimed to examine the acute effects of different nutritional and physical recovery strategies on exercise performance, muscle damage, and perceived fatigue and exertion in elite basketball players. Methods: Fifteen elite male basketball players participated in this pilot randomized crossover trial and completed four recovery conditions: cold-water immersion (CWI), active recovery (ACT), protein–carbohydrate supplementation (SUP), and placebo (PLA). Following a basketball-specific fatigue protocol, creatine kinase, countermovement jump performance, isometric strength, 10 m sprint, and 4 × 10 m shuttle run tests were assessed at baseline, immediately post-exercise, and 24 h post-exercise. Perceived fatigue and rate of perceived exertion were measured at baseline, immediately post-exercise, immediately after the recovery intervention, and 24 h post-exercise. Results: The three recovery methods attenuated the 24 h exercise-induced increase in CK compared with the placebo condition (*p* > 0.05). CWI, SUP and ACT decreased fatigue and RPE immediately after their application (*p* < 0.05), while PLA kept them elevated. CWI was associated with a significant improvement in 4 × 10 m SRT performance (*p* = 0.027). Conclusions: Nutritional supplementation and physical recovery strategies effectively attenuated exercise-induced muscle damage and fatigue in elite basketball players. However, CWI demonstrated the most pronounced acute benefits for physical performance recovery.

## 1. Introduction

Basketball is one of the most popular sports in the world [1]. It is a team sport in which five players face off against five others, with an indefinite number of changes that can be made when the coach deems it appropriate. It is a sport where anaerobic metabolism predominates and where the execution of technical actions at a high intensity is essential, as well as the ability to repeat these maximum efforts many times [2,3,4]. Competitive basketball involves frequent high-intensity efforts such as sprints, jumps, and rapid changes of direction, which lead to marked neuromuscular and metabolic fatigue over the course of training sessions and matches [2]. These high-intensity actions lead to a rapid accumulation of both central and peripheral fatigue within a short period of time [5].

In applied settings, recovery strategies are typically implemented following training sessions or competitive phases characterized by high external and internal loads, rather than being selected arbitrarily. These strategies are commonly integrated within periodized training programs to facilitate recovery from accumulated fatigue and to optimize subsequent performance [6,7]. In recent years, the elite basketball sport calendar has become condensed [8], with elite athletes having to play matches as often as every two or three days between national and international matches [8]. Therefore, this can have consequences for both physiological and psychological status and has the potential to impair performance [9]. Both the frequency of training sessions and matches and the physical demands required at high performance increase the risk of injury, despite the measures implemented during training to prevent these injuries [10].

These circumstances make recovery between training sessions and games essential [11]. Recovery must be effective, as fast as possible and without negatively interfering with the desired adaptations in the athlete or, at least, minimizing these interferences [12]. In this regard, there are many studies that have investigated the effects of different recovery methods and strategies in many sports and training types [4,13,14]. Specifically, in basketball, some of the most studied recovery strategies include cold-water immersion, carbohydrate–protein supplements, and active recovery, the most used method in basketball [4]. These methods have shown a high level of evidence and are the most frequently applied in relation to recovery effect. These recovery strategies have been shown to quickly recover physical performance and muscle damage [4]. However, to the best of the authors’ knowledge, no previous studies have analyzed the potential effect of simultaneously combining these above-mentioned methods in basketballers.

Thus, the main aim of this study was to examine the acute effects of different nutritional (carbohydrate–protein supplementation (SUP)) and physical recovery strategies (cold-water immersion (CWI) and active recovery (ACT)) on exercise performance, muscle damage, and perceived fatigue and exertion in elite basketball players.

## 2. Materials and Methods

### 2.1. Participants

A total of 15 elite male basketball players (mean ± standard deviation, age 23 ± 4 years) participated in the study. All players competed in the Spanish Amateur Basketball League (EBA league) of the Spanish Basketball Federation (FEB), specifically on the CB Cimbis team (San Fernando, Spain), and qualified to play in the promotion phase to the Silver Spanish Basketball League (LEB SILVER The 3^rd^ Division). All participants were elite male basketball players with extensive competitive experience at a national or professional level.

Due to the exploratory nature of the study and the elite population involved, no a priori power calculation was performed. The sample size was determined by the availability of eligible elite athletes. The general characteristics of the participants are described in Table 1.

The inclusion criteria were: (1) not having recently suffered injuries, (2) having at least 10 years of sports practice, (3) having been training during the season a minimum of four times a week, and (4) having competed and trained regularly in the previous season. The exclusion criteria were: (1) suffering an injury during the study period or (2) not having gone through all the treatment groups (CWI, SUP, ACT and PLA).

To minimize potential interference from dietary changes or the use of other nutritional supplements, participants were instructed to maintain their usual dietary intake throughout the study period and avoid the consumption of any dietary supplements that could potentially provide ergogenic benefits. Also, there were no restrictions (e.g., no exercise 24 h before the protocol visit, no caffeine for 8 h prior) prior to the study visits. The study visits occurred two day after the first training.

To track their training activities, participants completed a self-administered questionnaire detailing their weekly team practice duration and frequency of training.

The participants followed a similar diet established by the respective coaching staff of each of the participating clubs. Before the start of the study, it was determined that the participants were fit to play and train (i.e., that none had any injuries). None of the players had any injuries, allergies, or hormonal disturbances during data collection. In addition, none of the participants were under the influence of any illegal drugs or taking medication that affected body mass. The work performed during the training sessions was agreed on by the coaching staff and was therefore representative of the workload experienced during that period of the season [15].

### 2.2. Ethical Issues

After being informed about all the details of the experimental procedures and methods, including the potential risks of the study, each participant signed an informed consent form. It should be noted that the obtained data were treated with the greatest confidentiality and scientific rigor, with their use restricted according to the guidelines for research projects following the scientific method required in each case, in compliance with the Organic Law 15/1999 of 13^th^ December on the Protection of Personal Data (OLPPD); the procedures respected the ethical criteria of the Responsible Committee of Human Experimentation (established by law 14/2007, published in the Spanish Official State Gazette, n° 159). Ethical approval for the study was obtained from the ethical committee of the University of Cádiz (Puerta del Mar University Hospital, Registration Number: PEIBA 2123-N-21: 11.22), and the study was conducted in accordance with the Declaration of Helsinki [16].

### 2.3. Experimental Design and Procedures

**Figure 1:**
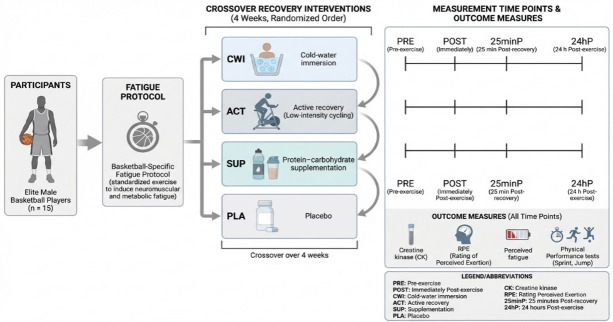
Overview of the experimental design and recovery protocols.

This study is part of The Recovery Project (NCT05805540A). Body composition, maximal oxygen consumption (VO_2max_) and maximal heart rate (HR_max_) were determined prior to the intervention. Then, a crossover design was used to examine the effects of 4 different recovery interventions on muscle damage, fatigue and physical performance after a fatigue exercise protocol in elite male basketball players, which included basketball-specific technical actions such as changes of direction or jumps [17]. Thus, each athlete performed all the recovery methods that were included in the study one week apart, including CWI (n = 15), SUP (n = 15), ACT (n = 15), and placebo control (PLA) (n = 15) conditions. The participants were randomly organized into four different groups by an independent statistician using OxMar open-source software (2019 version). Physical performance and biochemical markers of muscle damage were measured at baseline (PRE), immediately after the fatigue protocol (POST) and 24 h later (24 h). Furthermore, perceived exertion and fatigue were obtained at PRE, POST, immediately after the applied recovery method (25 min), and 24 h. The study was carried out at the end of the season (one week after the last league competition match), the moment in the season when athletes usually have the highest level of fatigue and when more injuries occur [18], allowing participants to focus completely on the study and thus avoiding confounding variables related to practice and match load. A schematic overview of the experimental design and recovery protocol is presented in Figure 1.

All participants kept two of their weekly team training sessions and did not play any matches during the intervention period. The weekly physical training session they had was replaced by the day they performed the study’s fatigue protocol. Players were instructed to eat and perform activities similarly to how they did during the first week of intervention. All participants belonged to the same team and followed an identical training schedule throughout the study period, with no differences in training frequency or volume between experimental conditions.

#### 2.3.1. Body Composition, Height and Cardiorespiratory Fitness

Before the beginning of the study, the participants were assessed at the Andalusian Sports Medicine Center (CAMD) in San Fernando (Cádiz, Spain), and body composition, VO_2max_ and HR_max_ were determined.

Body composition and height were assessed using a Tanita MC780MA (Tanita^®^, Tokyo, Japan) and a wall-mounted stadiometer (Tanita Leicester Portable^®^, Tanita Corp., Barcelona, Spain), respectively.

Participants performed a maximal incremental test on a treadmill for VO_2max_ and HR_max_ assessment. Following a 3 min warm-up period at a speed of 8 km/h, the speed was systematically increased by 1 km/h every three minutes until participants reached exhaustion. To simulate the physiological demands of outdoor running, a 1% gradient was applied to the treadmill [19], and measurements were monitored using an open-circuit gas analyzer, Jaeger MasterScreen CPX^®^ (CareFusion, San Diego, CA, USA), to determine VO_2max_ and HR_max_. The athletes were instructed not to consume alcohol or stimulants in the 24 h prior to the tests and to avoid vigorous physical exercise the day before.

#### 2.3.2. Recovery Methods

For the CWI condition, the participants were immersed in cold water at 11 °C for 2 min in a sitting position, with their legs fully covered, followed by 2 min out of the water in a sitting position at room temperature (25 °C); this process was repeated 5 times [4,20]. The SUP group consumed a mixed carbohydrate and protein beverage, composed of 0.3 g/kg of maltodextrin and 0.2 g/kg of neutral whey protein in 0.5 L of water [4]. The ACT condition was carried out by pedaling for 25 min at 50% of the HR_max_ on a cyclo-ergometer [21]. The PLA group drank a water-based drink and sweetener. Both the PLA and SUP groups were in a double-blind condition in a sitting position for 25 min until all recovery methods were completed.

#### 2.3.3. Exercise Protocol

The participants performed a basketball-specific exercise protocol with maximal effort until fatigue, which included the following specific basketball circuit: (a) backward shift in defensive position, (b) straight-line race with ball bounce and abrupt change of direction, (c) entry to the basket, (d) three jumps to the board, (e) slalom with ball bounce, (f) slalom without the ball between cones, simulating direct block defense action (3 to the right and 3 to the left), (g) backward sprint and (h) frontal sprint. This exercise protocol was specifically designed for the present study, being informed by previously published protocols and basketball match demands reported in the literature [17].

The athletes performed 5 sets of 5 repetitions, with 30 s of rest between repetitions and 1 min between sets. To avoid crowds in the rest areas and when performing post-effort tests, the start of the protocol was carried out in a staggered manner every 15 s. During the performance of the fatigue protocol, the heart rates of the athletes were monitored (Polar Team 2^®^, Polar, Kempele, Finland). In addition, after each series, to quantify the perceived exertion of participants, the Borg Perceived Exertion Scale was used. Heart rate peak (HR_p__eak_) and blood lactate concentration were measured to ensure that the exercise protocol was performed to the maximum by the participants of all groups.

#### 2.3.4. Outcomes

Creatine Kinase and Blood Lactate Concentration: Blood lactate concentration was measured to examine the metabolic stress induced by the fatigue protocol. Blood samples were obtained from the earlobe and collected into capillary tubes, and lactate was determined with Lactate Pro 2 (LT-1730, Arkray, Kyoto, Japan); creatine kinase (CK) activity was analyzed using a Reflotron^®^ Plus–Roche (Roche Diagnostics ^®^ SL, Barcelona, Spain) immediately after collection.

Fatigue and Rate of Perceived Exertion: Fatigue was assessed using a specific visual analog scale on a scale from 1 to 10 (1 = no fatigue and 10 = extremely fatigued) and rate of perceived exertion (RPE) was also measured using the 0–10 Borg Scale in each participant. The players filled out the questionnaires evaluating fatigue and RPE before and immediately after the exercise, after the recovery method application and 24 h later.

Performance Tests: Performance tests included a 10 m sprint test (10 mST), 4 × 10 m shuttle run test (4 × 10 mSRT), countermovement jump (CMJ), and isometric strength in half-squat exercise (IsoS). In the 10 mST, the players ran 10 m at maximal effort. In the 4 × 10 mSRT, the subjects had to run as fast as possible between two cones 10 m apart 4 times. The 10 mST and 4 × 10 mSRT times were recorded with video analysis. In CMJ [22], the participants were asked to keep their hands on their hips and jump as high as possible. Flight time was used to measure jump height and was measured on a force platform (Quattro Jump, Kistler, Winterthur, Switzerland). Jump height (h) was calculated from flight time (t) using the formula h = t^2^ × g/8, where g = 9.81 m·s^−2^. For IsoS, participants performed a maximal isometric contraction for 6 s in a half-squat exercise at 90° knee flexion, using a Smith machine (Nautilus NT 1800; Nautilus, Inc., Vancouver, WA, USA). The strength of the participants was measured with a force platform on which the exercise was performed. The players were instructed to exert maximal strength from the start and not to stop until given a signal. The participants had 2 attempts per test to achieve their best performance with 2–3 min of rest between trials. For the CMJ test, the participants had 3 attempts, and we used the best performance for the statistical analysis.

After the RPE, fatigue and CK measurements, and before the beginning of the physical performance tests, players ran for 5 min as a warm-up and performed 5 min of light stretching. Players were familiar with the procedures from the preseason and reported their fatigue perception and RPE every day before training and competitive sessions.

### 2.4. Statistical Analysis

Data are expressed as mean ± standard deviation (SD). The Shapiro–Wilk test (<50) was used to check the normality of data distribution, and the Levene test was used for homoscedasticity.

As the data followed a normal distribution, a 3 × 3 repeated-measure analysis of variance (ANOVA) was used to compare the evolution of physical performance variables over time. A 3 × 4 repeated-measure ANOVA was used for the mean difference in perceived fatigue and exertion, as an extra measurement was taken immediately after the recovery method application. Bonferroni post hoc comparisons were applied. Effect size (ES) by partial eta squared was included and was classified as follows: higher than 0.90 was very large, from 0.75 to 0.90 was large, from 0.5 to 0.75 was moderate, and less than 0.25 was small. In addition, 95% confidence intervals with corresponding *p*-values were included. A *p*-value < 0.05 was considered statistically significant. All analyses were performed using SPSS Software (version 25; SPSS Inc, Chicago, IL, USA).

A post hoc power analysis was conducted to estimate the statistical power of the main findings based on the actual sample size. Using the observed effect size for CK concentrations at 24 h post-exercise (Cohen’s d ≈ 0.55), an alpha level of 0.05, and a sample size of 15 participants, the achieved statistical power was approximately 0.65. This analysis was performed to allow readers to better contextualize the results of this pilot study.

## 3. Results

All exercise protocols performed in each recovery method showed the same fatigue, with similar blood lactate concentration, HR_peak_ and RPE levels (Table 2).

### 3.1. Time and Recovery Method Effects

A time main effect for CK (F1.551, 21.7 = 19.166; *p* < 0.001; ES = moderate), fatigue (F3, 42 = 41.471; *p* < 0.001; ES = moderate), RPE (F2.046, 28.642 = 169,931; *p* < 0.001; ES = very large), 4 × 10 mSRT (F2, 28 = 6.644; *p* = 0.004; ES = small) and IsoS (F2, 28 = 3.401; *p* = 0.048; ES = small) was observed. There was a main effect of the recovery method for 10 mST (F3, 42 = 5.028; *p* = 0.005; ES = small). There were no statistically significant interactions between recovery method and time for any variable. The POST compared with PRE measurements of CK, fatigue and RPE showed statistically significant differences (*p* < 0.05). Bonferroni post hoc comparisons are presented in Table 3.

#### 3.1.1. Cold-Water Immersion

For CK, there were differences between PRE and 24 h (*p* = 0.003), but not between POST and 24 h (*p* = 0.125) when athletes used CWI. Fatigue values were significantly lower from POST to 25 min (*p* < 0.001) with the use of CWI. 25 min levels were not significantly different to PRE (*p* = 0.082) or 24 h (*p* = 0.103). For RPE, there were significant decreases observed at 25 min compared with POST (*p* < 0.001), and 24 h after RPE was significantly lower than at 25 min (*p* = 0.035). The 4 × 10 agility test time improved significantly (*p* = 0.027) at 24 h compared with POST when CWI was used. There were no significant differences in CMJ height between measurement moments, but we observed a trend (*p* = 0.067) toward improvement 24 h after CWI application. There were no differences between moments (*p* > 0.05) for 10 mST or IsoS.

#### 3.1.2. Protein + Carbohydrate Supplement

For CK, there were differences between PRE and POST (*p* < 0.001) and PRE and 24 h (*p* = 0.014), but not between POST and 24 h (*p* = 0.100) when athletes used SUP. Fatigue values were significantly higher from PRE to POST (*p* < 0.001), and 25 min values were significantly lower (*p* = 0.044) than POST with the use of SUP. 25 min levels were not significantly different to PRE (*p* = 0.067) and 24 h (*p* = 0.072). RPE values in POST were significantly higher (*p* < 0.001) than PRE values in the SUP condition. There were significant decreases observed at 25 min compared with POST (*p* = 0.001), and at 24 h compared with 25 min (*p* = 0.004) when SUP was used. There were no significant differences in IsoS between measurement moments, but we observed a trend (*p* = 0.078) toward improvement 24 h after the SUP application. There were no differences between moments (*p* > 0.05) for 10 mST, 4 × 10 mSRT, or CMJ when SUP was used.

#### 3.1.3. Active Recovery

For CK, there were differences between PRE and POST (*p* = 0.001) and PRE and 24 h (*p* = 0.004), but not between POST and 24 h (*p* = 0.075) when athletes used ACT. Fatigue values were significantly higher from PRE to POST (*p* = 0.004), and 25 min values were significantly lower (*p* = 0.015) than POST with the use of ACT. 25 min levels were not significantly different to PRE (*p* = 0.157) and 24 h (*p* > 0.05). The RPE values in POST were significantly higher (*p* < 0.001) than PRE values in the ACT condition. There were significant decreases observed at 25 min compared with POST (*p* < 0.001) and at 24 h compared with 25 min (*p* = 0.001) when ACT was used. There were no differences between moments (*p* > 0.05) for 10 mST, 4 × 10 mSRT, CMJ, or IsoS when ACT was used.

#### 3.1.4. Placebo

For CK, there were significant differences between all moments (*p* < 0.05) when athletes used PLA. Fatigue values were significantly higher from PRE to POST (*p* < 0.001). 25 min values were not significantly different (*p* = 0.159) from POST with the use of PLA. 25 min levels were significantly higher than PRE (*p* = 0.006) and 24 h (*p* = 0.017). The RPE values in POST were significantly higher (*p* < 0.001) than PRE values in the PLA condition. There were significant decreases observed at 25 min compared with POST (*p* < 0.001), and at 24 h compared with 25 min (*p* = 0.008) when PLA was used. PRE rate of perceived exertion levels were similar to those at 24 h (*p* > 0.05).

## 4. Discussion

The main findings of this study were that CWI appeared to be the most effective recovery method among those examined within the context of this pilot trial. This method presents the effectiveness in attenuating the increase in blood CK after 24 h of recovery, with a positive effect when ACT and SUP were used. In addition, all three recovery methods (CWI, SUP, and ACT) were effective in immediately improving perceived fatigue and RPE condition, while the PLA condition did not show this improvement. Regarding physical performance, CWI was the only recovery strategy that resulted in a statistically significant improvement in 4 × 10 m shuttle run performance 24 h post-exercise. Furthermore, non-significant trends toward improved 10 m sprint and CMJ performance were observed following CWI. Similarly, SUP showed a non-significant trend toward improved isometric strength recovery at 24 h. These trends did not reach statistical significance and should therefore be interpreted with caution.

The positive effects of CWI on CK have been observed in previous studies performed on male athletes of different team sports [6,23]. Specifically, CWI may improve recovery from muscle damage in professional basketball players during a regular season [24]. Proposed mechanisms include a reduction in edema through decreased blood flow, which may facilitate the clearance of accumulated metabolites, as well as cold-induced vasoconstriction that increases venous pressure and may contribute to metabolite removal. In addition, reductions in intramuscular temperature may decrease metabolic activity and limit secondary muscle damage [25]. However, evidence regarding the effectiveness of CWI in reducing CK concentrations 24 h after intense exercise remains conflicting [26,27]. Some studies have reported delayed effects, with reductions in CK observed at later time points (e.g., 48 h post-exercise) rather than at 24 h [14]. The time from exercise to CK measurement seems to clarify the effects that CWI could have on CK. Therefore, more research should be carried out comparing the effects of this recovery method on the evolution of CK at 24, 48, and 72 h, specifically in basketball.

SUP use has been shown to attenuate the release of CK into the blood after physical exercise in our pilot study. In line with these results, a study which included strength and power athletes observed this attenuation of the release of CK using SUP compared with PLA [28]. Furthermore, a recent review [29] concluded that, in the long term, sustained consumption of SUP could decrease the release of CK into the bloodstream. Nevertheless, evidence also suggests that protein-based supplements, including those combined with carbohydrates, may attenuate CK responses in the short term without necessarily leading to significant improvements in physical performance [29,30]. In this same study, the authors observed that these effects of SUP on CK would not be related to a significant improvement in physical performance [29].

The effects of active recovery on post-exercise CK levels remain controversial. While some studies report that low-intensity exercise performed after training or competition may enhance CK clearance [31], other investigations have shown limited effectiveness [32,33]. A recent systematic review with meta-analysis [6], which included 43 studies, concluded that ACT is an effective method to reduce CK release 24 h after exercise, and therefore to reduce exercise-derived muscle damage. However, the variability in ACT protocols across studies complicates direct comparisons. Future research should examine the influence of different ACT modalities, intensities, and durations to better determine the conditions under which this recovery strategy may be most effective.

Concerning fatigue and RPE variables, in our pilot study, we observed that CWI, SUP, and ACT were effective in recovering fatigue perception and RPE, CWI being the recovery method that reduce the fatigue and RPE to basal values more quickly (immediately after application). There is strong scientific evidence supporting our results regarding the effects of CWI use on the perception of fatigue and RPE [34,35]. However, there are conflicting results related to the effects on perceived fatigue when ACT is used. The use of ACT may not be the best post-exercise strategy to reduce the perception of fatigue [6]. In particular, most studies show that the use of ACT does not have positive effects on the perception of fatigue; however, this method could be better than passive recovery [7]. In this study, the best or worst response of ACT versus passive recovery depends on the athletes’ level. Concretely, for amateur athletes, performing an ACT involves extra effort and they perceive it as doing more physical exercise, while for professional athletes, ACT seems to be better than passive recovery. In line with these results, a systematic review [36] concluded that only short-duration ACT protocols (6–10 min) appear effective for improving perceived recovery [36], whereas the ACT duration used in the present study was longer (25 min).

To our knowledge, our study is the first to specifically investigate the effects of SUP on the subjective perception of fatigue in basketball. Therefore, although these results provide us with new knowledge and strategies on how to improve our athletes’ perception of fatigue, further studies are required to confirm and contextualize these results in different basketball populations and competitive settings.

Regarding the physical performance variables of the present study, CWI improved the performance in the 4 × 10 mSRT test and showed a non-significant trend toward improvement in the 10 mST, without significant effects of SUP, ACT and PLA for these variables. Although there were no significant differences in 10 mST time between moments of measurement for any recovery method (*p* > 0.05), when PLA and ACT were used, the 10 mST time showed clinical increments 24 h later, whereas when using CWI and SUP this time decreased. The effectiveness of recovery using CWI on sprint performance has been observed in some studies and there are a few studies performed in team sport athletes (some of them in basketball players) in which the effects of CWI recovery were examined on agility performance [34,35,37]. Related to these tests, we found that the use of CWI after intense exercise is effective in accelerating the recovery of leg power [20], which theoretically would favor performance in the sprint and agility tests [38], two predominant physical skills in elite basketball players [2].

For the recovery of the CMJ performance, CWI was the only recovery method that showed a non-significant positive trend, with a trend toward significant differences after 24 h with the use of CWI. The non-significant trend shown by our results is consistent with current evidence, which shows that CWI is effective in reducing performance losses derived from muscle fatigue in CMJ in team sports players [23,34,35] and specifically in basketball players [37]. In line with our results, another study [23] showed that there are no differences in this regard between ACT and CWI, although the effectiveness of ACT on performance recovery in CMJ is not entirely clear. A review [7] observed that the effects of the use of ACT on the CMJ performance the day after were small or moderate. In our study, SUP had no beneficial effects on CMJ recovery after 24 h, without differences with the PLA condition, something that had already been seen in previous studies performed on different team sports [29].

For IsoS, no statistically significant positive effects of any recovery method were seen after 24 h. In the case of CWI, the results of other studies showed results like ours [20,39]. Although it did not show a statistically significant improvement, the use of SUP showed a non-significant trend toward improving IsoS performance 24 h after exercise to fatigue. No studies were found specifically investigating the effects of SUP after 24 h on maximal strength. In high-intensity exercise, the availability of carbohydrates in the body is a key factor to obtain maximum performance [40]. We know that SUP after intense physical exercise is effective in restoring the body’s glycogen stores [41]. Therefore, in line with our results, the use of SUP could have positive short-term effects on the recovery of strength, but more well-designed studies are needed to clarify this issue. Regarding the effects of ACT on strength, there is evidence that this recovery method is not effective in improving muscle strength 24 h after strenuous exertion. This may be because it was observed that this method was effective in accelerating recovery at the central level but not the contractile properties of the muscle, without beneficial effects on the maximum voluntary contraction [37]. Furthermore, it was observed that ACT is not effective in improving physical performance in general, and strength particularly, 24 h after its application. More studies are needed to observe the effects of this recovery method after different periods of time (24 h, 48 h, 72 h, at least), based on individualization [42].

From an applied perspective, the findings of this pilot study suggest that CWI may be particularly useful following training sessions or matches characterized by high neuromuscular and metabolic demands, especially when rapid recovery is required within 24 h. Based on the protocol used in the present pilot study, practitioners may consider applying CWI using short immersion bouts (e.g., repeated cycles of 2 min immersion with brief rest periods) in the immediate post-exercise phase. Regarding active recovery and protein–carbohydrate supplementation, these may also be useful strategies to reduce perceived fatigue and exertion, particularly when implemented shortly after exercise. However, given the pilot nature of the study, the optimal frequency and long-term application of these recovery strategies should be individualized and further investigated in future research.

Our study is not free of limitations and, therefore, further research is needed to complement the results provided. First, the relatively small sample size, inherent to studies involving elite athletes, limits the generalizability of the findings and reduces statistical power to detect small-to-moderate effects. Consequently, longitudinal studies are needed to determine the stability and sustainability of these effects over time, and future research should compare these recovery methods with other emerging strategies, such as blood flow restriction. In addition, the exclusive inclusion of male high-performance basketball players limits the extrapolation of the results to female athletes, other competitive levels, or sports with different physical and physiological demands. Finally, the use of a convenience, non-probabilistic sampling approach may further limit representativeness. Nevertheless, this study was designed as a pilot investigation to provide exploratory evidence in an elite athletic context rather than to generalize findings to the general population.

In addition to these limitations, our study has several strengths. The randomized crossover design of this study increases the internal validity. The inclusion of a placebo condition as a control also increases the internal validity, allowing us to compare the recovery methods with this group. Another strength of our study is the double-blind design, in which the evaluators did not know the recovery method that had been applied to each participant, and participants were unaware if they were taking SUP or PLA, avoiding bias in this regard.

## 5. Conclusions

Our findings suggest that nutritional supplementation and physical recovery strategies may attenuate exercise-induced muscle damage and perceived fatigue in elite basket-ball players. Among the strategies examined, our results mainly showed that CWI appeared to be the most effective method in our study for reducing exercise-derived muscle damage, measured by creatine kinase, which can lead to a significant improvement in sprint, agility, and countermovement jump performance.

Additionally, consuming a carbohydrate supplement mixed with protein and utilizing active recovery after physical exertion could have beneficial effects on perceived fatigue and positively impact isometric strength recovery. However, given the pilot nature of the study and the limited sample size, these findings should be interpreted with caution and warrant confirmation in future adequately powered investigations.

## Figures and Tables

**Table 1:** Descriptives statistics of the sample (n = 15) of elite male basketball players.

Variable	Mean (SD)	Minimum	Maximum
Age (years)	22.13 (3.66)	18.00	32.00
Body mass (kg)	93.35 (16.31)	72.50	125.30
Height (cm)	192.99 (5.93)	183.40	205.50
BMI (kg/cm^2^)	25.01 (3.82)	19.14	32.29
Body fat percentage (%)	10.14 (3.50)	5.98	19.09
VO_2max_ (mL/min)	45.83 (4.72)	40.40	54.80
Heart rate max (bpm)	184.87 (9.73)	172.00	201.00

Abbreviations: BMI = body mass index; bpm = beats per minute; SD = standard deviation.

**Table 2:** Values of maximum effort and accumulated fatigue in the basketball-specific fatigue protocol for each recovery method.

Variable	PLA (n = 15)	CWI (n = 15)	ACT (n = 15)	SUP (n = 15)	*p*
Post-Exercise Lactate (mmol/L)	3.99 (1.58)	3.88 (1.93)	3.62 (2.08)	4.19 (1.90)	0.866
RPE (0–10 scale)	8.13 (1.88)	8.47 (1.36)	8.60 (1.40)	8.40 (1.84)	0.886
Fatigue Protocol HR_peak_ (bpm)	186.87 (8.65)	188.93 (8.36)	187.60 (8.16)	188.40 (7.86)	0.909

Data are presented as the mean (standard deviation at 95% CI). *p*-values indicate significant differences between conditions. Significance was set at *p* < 0.05. Abbreviations: PLA, placebo; CWI, cold-water immersion; SUP, carbohydrates and protein co-ingestion; ACT, active recovery; RPE, rate of perceived exertion; HR_peak_, heart rate peak.

**Table 3:** Post-recovery changes in rate of perceived exertion, fatigue, muscle damage and neuromuscular and physical performance.

Variable	Placebo (n = 15)	Cold-Water Immersion (n = 15)	Active Recovery (n = 15)	CHO–PRO Supplement (n = 15)
CK (mmol/L)				
PRE	85.09 (71.73)	61.00 (18.25)	72.29 (30.18)	68.41 (30.77)
POST	110.50 (85.51) *	85.93 (27.03) *	95.89 (34.74) *	94.09 (36.27) *
24 hP	163.11 (85.18) &	122.68 (57.91) *	142.11 (71.51) *	136.15 (77.54) *
RPE (0–10 scale)				
PRE	0.53 (1.36)	0.13 (0.52)	0.47 (1.25)	0.07 (0.26)
POST	8.13 (1.89) *	8.60 (1.40) *	8.47 (1.36) *	8.40 (1.84) *
25 minP	3.13 (2.42) &	2.07 (2.66)	4.13 (2.64) &	3.67 (3.13) &
24 hP	0.67 (1.35) #	0.40 (1.06) #	0.60 (0.91) #	0.53 (1.19) #
Fatigue (0–10 scale)				
PRE	1.35 (1.59)	1.72 (1.65)	1.92 (1.95)	1.59 (1.83)
POST	4.95 (1.79) *	5.97 (2.01) *	4.51 (2.61) *	5.12 (2.58) *
25 minP	3.85 (2.04)	3.04 (1.39) $	2.93 (1.92) $	3.49 (2.15) $
24 hP	1.93 (1.81) #	1.85 (2.19) $	2.73 (2.30) $	1.94 (1.83) $
10 mST (s)				
PRE	2.019 (0.195)	2.047 (0.169)	2.103 (0.159)	1.980 (0.134)
POST	2.049 (0.157)	2.077 (0.193)	2.111 (0.156)	1.999 (0.133)
24 hP	2.055 (0.129)	1.990 (0.136)	2.136 (0.143)	1.973 (0.132)
4 × 10 mSRT (s)				
PRE	10.103 (0.450)	10.065 (0.486)	10.123 (0.455)	10.063 (0.507)
POST	10.139 (0.518)	10.131 (0.540)	10.139 (0.474)	10.142 (0.561)
24 hP	10.021 (0.459)	9.960 (0.497) $	10.047 (0.458)	10.054 (0.556)
CMJ height (cm)				
PRE	33.31 (4.13)	34.18 (3.64)	32.97 (5.21)	33.34 (3.61)
POST	34.23 (3.71)	32.35 (3.41)	32.94 (4.48)	32.92 (3.47)
24 hP	33.65 (5.66)	33.42 (3.59)	33.81 (4.62)	32.49 (3.74)
Isometric strength (kg)				
PRE	208.46 (50.51)	208.61 (41.44)	213.69 (44.93)	212.80 (35.98)
POST	195.45 (35.04)	189.91 (69.41)	203.78 (48.95)	200.96 (35.10)
24 hP	203.37 (42.10)	188.05 (37.03)	187.97 (71.32)	212.30 (33.24)

Data are presented as mean (standard deviation). Abbreviations: CK, creatine kinase; CMJ, countermovement jump; RPE, Rate of Perceived Exertion; 10 mST, 10 m sprint test; 4 × 10 mSRT, 4 × 10 m shuttle run test; 25 minP, immediately post-recovery method measurement; 24 hP, 24 h post-exercise values. Statistical significance: * shows *p* < 0.05 vs. PRE within the same condition; $ shows *p* < 0.05 vs. POST within the same condition; & shows *p* < 0.05 vs. PRE and POST within the same condition; # shows *p* < 0.05 vs. POST and 25 minP within the same condition.

## Data Availability

The original contributions presented in the study are included in the article. Further inquiries can be directed to the corresponding author.
